# Venous thromboembolism in in-hospital cirrhotic patients: A systematic review

**DOI:** 10.3389/fmed.2022.1027882

**Published:** 2022-11-07

**Authors:** Leonardo da Cruz Renó, Francisco Tustumi, Daniel Reis Waisberg, Vinicius Rocha-Santos, Rafael Soares Pinheiro, Rubens Arantes Macedo, Lucas Souto Nacif, Liliana Ducatti, Rodrigo Bronze De Martino, Alexandre Maximiliano Trevisan, Luiz Carneiro-D’Albuquerque, Wellington Andraus

**Affiliations:** Transplantation Unit, Department of Gastroenterology, Universidade de São Paulo, São Paulo, Brazil

**Keywords:** liver cirrhosis, venous thrombosis, systematic review, liver, bleeding

## Abstract

**Introduction:**

Patients with liver cirrhosis are at a higher risk of hospitalization. The present review aimed to assess the risk of thromboembolism and its burden on hospitalized cirrhotic patients.

**Materials and methods:**

A systematic review (PROSPERO: CRD42021256869) was conducted in PubMed, Embase, Cochrane, Lilacs, and a manual search of references. It evaluated studies that compare cirrhotic patients with venous thromboembolism (VTE) with cirrhotic patients without VTE or studies that compare cirrhotic patients with non-cirrhotic patients. No restrictions were set for the date of publication or language. The last search was conducted in June 2021.

**Results:**

After selection, 17 studies were included from an initial search of 5,323 articles. The chronic liver disease etiologies comprise viral, alcohol, autoimmune, NASH (non-alcoholic steatohepatitis), cryptogenic, hemochromatosis, cholestasis, and drug-related. The included studies were conflicted regarding the outcomes of VTE, pulmonary embolism, or bleeding. Patients with cirrhosis associated with VTE had prolonged length of hospital stay, and patients with cirrhosis were at higher risk of portal thrombosis.

**Conclusion:**

In-hospital cirrhotic patients are a heterogeneous group of patients that may present both thrombosis and bleeding risk. Clinicians should take extra caution to apply both prophylactic and therapeutic anticoagulation strategies.

**Systematic review registration:**

[https://www.crd.york.ac.uk/PROSPERO/], identifier [CRD42021256869].

## Introduction

Patients with liver cirrhosis are at a higher risk of hospitalization (1). Several cirrhosis decompensations may demand in-hospital care, such as encephalopathy, ascites, infection, and bleeding (2).

Hospitalized patients usually require prophylactic anticoagulation to avoid thromboembolic events (3). However, clinicians frequently avoid using anticoagulation therapy in cirrhotic patients due to heterogeneity in pharmacokinetics in cirrhosis and safety concerns (4). The major reason that clinicians avoid using anticoagulation is the increased risk of bleeding. Cirrhotic patients lack procoagulant factors, such as fibrinogen, vitamin K, protein C, and platelets (4).

However, the risk of bleeding may lead clinicians to underestimate the risk of venous thromboembolism (VTE) in cirrhotic patients. The levels of antithrombin III, protein C, and protein S are significantly lower in patients with cirrhosis compared to controls without cirrhosis (4). The reduced levels of these anticoagulant factors may also make these patients more susceptible to VTE (4). The severity of the deficiency of these factors rises proportionally with Child-Pugh score (4). Increased levels of D-dimer and antiphospholipid antibodies in some types of chronic liver diseases may also contribute to the hypercoagulable state (4–6).

For non-cirrhotic patients, the population-attributable risk for thrombosis related to in-hospital immobility can reach over 25% (7). Prophylactic anticoagulation promotes an absolute risk reduction for fatal thrombosis of 0.25% in non-cirrhotic hospitalized patients (8). In spite of the known bleeding risks associated with anticoagulation drugs, anticoagulant prophylaxis has no significant increased risk for major bleeding in non-cirrhotic patients (8). However, the risk of VTE in cirrhotic patients is not fully defined, and their bleeding risk during hospitalization still needs more understanding.

The present review aimed to assess the risk of thromboembolism and its burden in in-hospital cirrhotic patients. The primary objective of this review was to compare the risk for VTE in in-hospital cirrhotic patients with in-hospital non-cirrhotic patients. As a secondary objective, we aimed to compare the outcomes of hospitalized cirrhotic patient complicated with VTE *vs*. hospitalized cirrhotic patient with no VTE.

## Materials and methods

This work was guided by the PRISMA statement (9) and was registered at the International Prospective Register of Systematic Reviews (PROSPERO: CRD42021256869).

### Database search

A systematic review was conducted in PubMed, Embase, Cochrane, Lilacs, and a manual search of references. No restrictions were set for the date of publication or language. The last search was conducted in June 2021. For PubMed, the search strategy used was [(pulmonary) OR (deep-vein) OR (deep-venous) OR (deep vein) OR (deep venous)] AND (thrombosis OR thromboses OR embolism OR emboli OR embolus OR thromboembolism OR thromboembolic OR phlebothrombosis OR thrombus OR thrombi) AND (liver OR hepatic) AND (cirrhosis OR cirrhotic OR fibrosis OR fibrotic).

Similar searches were performed in the other databases.

### Study selection

The inclusion criteria were as follows: (a) studies that compare cirrhotic patients with venous thromboembolism (VTE) with cirrhotic patients without VTE or studies that compare cirrhotic patients with non-cirrhotic patients; (b) hospitalized patients; (c) studies that evaluate the outcomes length of hospital stay (LOS), risk of VTE, risk of pulmonary embolism, risk of portal thrombosis, or risk of bleeding; and (d) cohort or case–control studies. The exclusion criteria were as follows: (a) case reports, reviews, letters, editorials, congress abstracts, and full-text unavailability. Two reviewers (LC and FT) searched and selected the articles using the previously defined eligibility criteria.

### Outcomes

The following in-hospital patients’ outcomes were assessed: length of hospital stay (LOS), risk of VTE, risk of pulmonary embolism, risk of portal thrombosis, or risk of bleeding.

### Analysis of bias and certainty assessment

The Risk of Bias in Non-Randomized Studies of Interventions (ROBINS-I) tool (10) was used for bias assessment. The Grading of Recommendations, Assessment, Development, and Evaluations (GRADE)^1^ was used for certainty assessment.

### Data extraction

Two researchers (LC and FT) extracted the following data: baseline characteristics of the included studies (cause of liver disease, sex, age, Child-Pugh, MELD, malignancy association with hospitalization, and infection); and the outcomes were length of hospital stay (LOS); venous thromboembolism (VTE) risk; pulmonary embolism risk; portal thrombosis risk; and bleeding.

### Data synthesis

Due to the high clinical heterogeneity of the included studies, with heterogeneous patient datasets, a meta-analysis was not suitable, and a qualitative synthesis was used.

## Results

**FIGURE 1:**
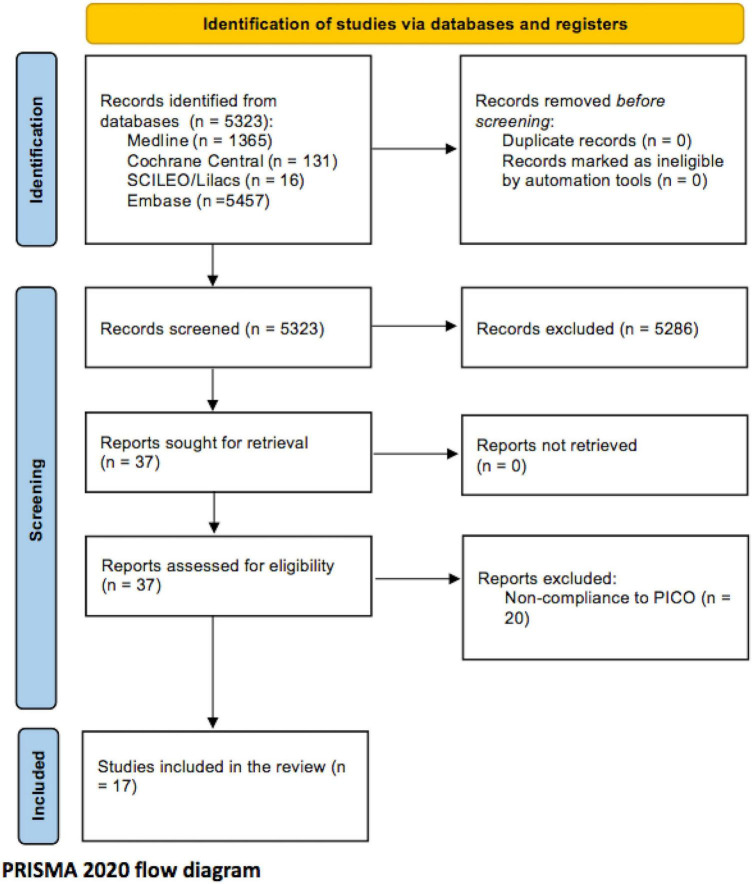
Selection flow diagram.

After selection, 17 studies (11–28) were finally included from an initial search of 5,323 articles. The systematic review comprised seven studies that compared cirrhotic patients with non-cirrhotic patients and 11 studies that compared cirrhotic patients with venous thromboembolism (VTE) and cirrhotic without VTE. Of the 17 studies, 6 were matched observational controlled studies. Figure 1 shows the PRISMA flow diagram for the study selection.

### Baseline characteristics

The mean age between the studied groups ranged from 49 to 73 years old. There was a predominance of males in the majority of the studies. Eight of the studies reported hospitalization due to hepatocellular cancer or any malignancy related to liver cirrhosis. There was a significant heterogeneity regarding baseline patients’ conditions. The included studies comprised different chronic liver disease etiologies, such as viral, alcohol, autoimmune, NASH, cryptogenic, hemochromatosis, cholestasis, and drug-related. Besides, the liver function status was heterogeneous among studies, and some of them did not report the Child-Pugh and MELD scores.

Supplementary materials 1, 2 present the baseline characteristics of the included studies.

### Venous thromboembolism

Enger et al. (11), in a matched-cohort study, detected a significantly increased incidence rate of VTE in the cirrhotic cohort (73.7 per 10,000 person-years) compared with the non-cirrhotic control (13.9 per 10,000 person-years), with a crude incidence rate ratio of 5.31 (95% CI 4.21 to 6.71). Ng et al. (12) performed a multivariate regression to evaluate the hazard of VTE, controlling the covariates age, sex, urbanization level, and comorbidities. Even after adjusted hazard evaluation, the cumulative risk of VTE remained higher in patients with liver cirrhosis (1.71; 95% CI 1.05 to 2.78). Wu and Nguyen (13) found a VTE occurrence rate of 7.6, 8.1, and 8.2 per 1,000 patient discharges, respectively, for patients with liver disease, compensated cirrhosis, and decompensated cirrhosis (*p* = 0.001). Yang et al. (14) found that chronic liver disease imposes significantly higher occurrence of overall VTE compared with that of non-chronic liver disease controls (risk of controls: 0.8%). This increase was seen both in the non-cirrhosis group with chronic liver disease (risk: 1.5%) and the cirrhosis group (risk: 2%).

Gulley et al. (15) also identified VTE risk difference between cirrhotic and non-cirrhotic patients only in univariate analysis. The presence of cirrhosis was not significant in the multivariate regression model after controlling for the covariates Carlson index, hemoglobin level, albumin level, and coagulation parameters. In Al-Dorzi et al.’s study (16), the risk for VTE was similar between cirrhotic and non-cirrhotic patients (2.7 vs. 7.6%, *p* = 0.11). Barba et al. (20), using a nationwide in-patient database in Spain, with 5,618,687 patients with no liver disease, 188,244 with mild liver disease, and 135,832 moderate–severe liver disease, suggested that VTE occurs in less than 2% of patients with chronic liver disease during hospitalization. Barba et al. (20) stated that the risk of VTE decreases (OR 0.397; CI 95%: 0.373 to 0.424) with the presence of moderate and severe liver disease. Dabbagh et al. (17) analyzed all patients admitted with chronic liver disease over 7 years, and in 190 cases, 12 developed VTE (6.3% incidence). They divided these patients into quartiles according to INR (international normalized ratio) values and demonstrated VTE occurs despite higher INR values. Girleanu et al. (18) over 2 years of observation divided patients with chronic liver diseases into two groups, liver diseases with and without VTE, including portal thrombosis. The 3,018 cases showed 0.99% incidence of VTE and 1.5% incidence or portal thrombosis. VTE cases showed lower levels of albumin and higher values of MELD (Model for End-Stage Liver Disease) scores and platelet counts. Shun Kohsaka el al. (19) analyzed 719 medical records of CLD (chronic liver disease) and found 10 patients with VTE at an incidence of 1.4%. They also showed higher platelet counts and INR values in VTE cases.

### Pulmonary embolism

Four studies evaluated the incidence of pulmonary embolism in hospitalized patients. The results were inconsistent among the studies.

Yang et al. (14), in a study with 1,296 patients, found that the risk of pulmonary embolism was higher for patients with chronic liver disease (0.6% vs. 0.3%, all *p* < 0.001) compared with non-chronic liver disease controls. Enger et al. (11) also stated that the incidence of pulmonary embolism was higher for the cirrhotic cohort (15 per 10,000 person-years) than the non-liver disease control group (5.9 per 10,000 person-years), with an incidence rate ratio of 2.73 (95% CI 1.7 to 4.38). The study included 15,158 patients.

However, Al-Dorzi et al. (16) analyzed data from 75 patients, comparing patients with cirrhosis and patients with no liver disease. The authors detected no significant difference for the risk of pulmonary embolism between the studied groups (0 vs. 3.3%; *p* = 0.11). Barba et al. (20) found that hospitalized patients with liver disease had a lower risk of pulmonary embolism, and this risk difference was more prominent in moderate and severe liver disease. The risk for pulmonary embolism in in-hospital patients with no liver disease was 1.6%, in cirrhotic patients was 1.3% for mild liver disease, and 0.4% for severe–moderate liver disease. The study included 135,832 patients.

### Acute portal thrombosis

Two studies evaluated the risk of portal thrombosis. Both studies compared hospitalized cirrhotic patients with hospitalized patients with no liver disease. Enger et al. (11) found that the incidence rate of acute portal thrombosis in patients with cirrhosis was 43.1 per 10,000 person-years, while in the comparator cohort the incidence was 0.2 per 10,000 person-years. Barba et al. (20) showed that the risk for portal thrombosis was dependent on the severity of the liver disease. Patients with no liver disease had a 0.1% risk, patients with mild liver disease had a 0.4% risk, and finally, patients with moderate–severe liver disease had a 2.3% risk.

### Bleeding

Seven studies evaluated the risk for variceal bleeding in hospitalized cirrhotic patients. The results were conflicting.

Barba et al. (20) evaluated the burden of VTE in hospitalized patients with liver disease. Patients with liver disease and VTE had higher odds of variceal bleeding than patients without VTE [2.2 vs. 0.5%; OR (odds ratio): 0.22; IC 95% 0.16 to 0.29]. Barba et al. (20) found that the risk for variceal bleeding was proportional to the severity of the liver disease. Patients with no liver disease were at low risk of variceal bleeding (0.01%). Patients with mild liver disease had a risk of 0.5%, and moderate–severe liver disease had a risk of 4.5%.

Bogari et al. (21) compared chronic liver disease patients with VTE and without VTE, in 145 patients. The risk for bleeding was significantly higher in those with VTE (16.7 vs. 1.4; *p* = 0.01). Bikdeli et al. (22) showed that the 1-year cumulative incidence of fatal bleeding was higher in those with cirrhosis, compared with patients without cirrhosis (32.4 vs. 11.3%). In addition, Bikdeli et al. (22) showed that patients with cirrhosis had a higher rate of 30-day fatal bleeding during the anticoagulation therapy (2.1 vs. 0.16%; *P* < 0.001).

Stine et al. (23) found no difference between gastroesophageal varices between patients with liver disease and VTE and patients without VTE (52.8 vs. 51%; *p* = 0.767) in 145 patients. Walsh et al. (24) also found no significant gastrointestinal bleeding risk comparing cirrhotic patients with VTE than in cirrhotic without VTE (25.9 vs. 22.2%) in 108 patients. However, the small sample size (27 with VTE and 81 without VTE) may have led to type-II error. Aldawood et al. (25) also evaluated a small sample size (six patients with VTE and cirrhosis) and found no significant difference for bleeding risks (33 vs. 23.6%; *p* = 0.63).

Ali et al. (26), in a population-based study from the Nationwide Inpatient Sample, surprisingly found a lower risk for variceal bleeding for patients with cirrhosis and VTE than patients with cirrhosis without VTE (6 vs. 8.6%, *p* = 0.001). However, in Ali et al.’s (26) study, patients with cirrhosis but without VTE had a baseline coagulopathy more often than the VTE patients (5.9 vs. 9.3%, *p* < 0.001), which could explain the higher risk of bleeding in the group without VTE. A total of 441.551 patients were studied.

### Length of hospital stay

Barba et al. (20) (11.8 vs. 10.4 days), Aldawood et al. (25) (43 vs. 8 days), Walsh et al. (24) (9 vs. 5 days), and Bogari et al. (21) (14.7 vs. 7.1 days) showed that hospitalized patients with liver disease and VTE had a longer length of hospital stay than patients with cirrhosis but without VTE.

Wu and Nguyen (13) evaluated the subgroups of compensated and decompensated cirrhotic patients in 649,879 patients. The authors found that the mean length of stay was longer for patients with VTE, both in the compensated cirrhosis subgroup (14.4 vs. 6.5 days) and in the decompensated cirrhosis subgroup (14.9 vs. 7.4 days). The VTE was associated with a 103% increase in the length of hospital stay among patients in the compensated cirrhosis subgroup and an 86% increase in the length of hospital stay in the decompensated cirrhosis subgroup.

### Mortality

Zhang et al. (27) showed that cirrhosis patients with VTE had a higher risk of in-hospital mortality than cirrhosis without VTE (33.3 vs. 3.4%). Wu and Nguyen (13) showed that the in-hospital mortality in patients with no liver disease and VTE was 9.8%. In patients with compensated cirrhosis and VTE, the risk of mortality was 16.8%, and in the decompensated cirrhosis group, the risk was 18.6%.

Barba et al. (20) found that the increased risk for mortality was evident only for moderate and severe liver disease. The risk of death with mild liver disease was 5.8%, and with moderate–severe liver disease was 13.9%.

Aldawood et al. (25) (66.6 vs. 33.2%; *p* = 0.18), Walsh et al. (24), (18.5 vs. 13.6%; *p* = 0.53), and Bogari et al. (21) (27.8 vs. 11%; *p* = 0.06) found no significant difference for hospital mortality. These three studies had small sample sizes.

Sogaard et al. (28) showed a higher 30-day mortality risk for patients with chronic liver disease and VTE compared with VTE without CLD. The CLD plus VTE group had 7% mortality risk, and the VTE group had 3%.

### Certainty assessment and risk of bias

The Robins-I risk of bias assessment tool showed that the major concern of the included studies relies on the risk of selection bias in the non-matched studies (see Supplementary material 3). For the certainty assessment, the primary concerns were the risk of bias and the inconsistency of outcomes (see Supplementary material 4).

## Discussion

This review showed high heterogeneity regarding VTE risk in outcomes of hospitalized patients with cirrhosis. Part of the outcomes heterogeneity is due to the variability of the baseline characteristics of the included studies, with a high number of liver cirrhosis etiologies and different grades of disease severity. Nevertheless, patients with liver cirrhosis sit inside a wide spectrum, ranging from a higher risk of clot formation to a higher risk of bleeding. Consequently, treatment should be tailored, and clinicians should evaluate patients’ factors associated with clotting against the variables associated with bleeding.

In patients with liver disease, the level of anticoagulant factors is reduced, meaning that patients are susceptible to VTE (4). However, hepatocytes are involved in the synthesis of most blood coagulation factors, such as prothrombin, fibrinogen, factors XII, XI, X, IX, VII, and V, proteins C and S, and antithrombin (29). Intra-hepatic endothelial cells also produce von Willebrand factor and factor VIII (29). Consequently, coagulation factors produced in the liver start to reduce according to the progression of liver disease or according to decompensating hepatic events. At this point, patients tend toward the other side of this clot-to-bleed spectrum. In addition to the lack of coagulation factors, patients with advanced liver disease usually present portal hypertension and esophageal varices, which represent a risk of bleeding on its own, even with normal coagulation level factors (30). These facts could explain why Barba et al. (20) found that the risk of VTE and pulmonary embolism decreases with the presence of moderate and severe liver disease. Also, Gulley et al. (15) show no difference between VTE risk for cirrhotic and non-cirrhotic patients when adjusting for the covariates such as albumin level and coagulation parameters, which comprise part of liver function tests.

Portal thrombosis formation occurs due to physiological mechanisms that are somewhat different to other types of thrombosis. In addition to the coagulation dysfunction seen in chronic liver disease patients, the stasis due to reduced portal vein flow velocity may also influence the risk of portal thrombosis (31). Cirrhotic patients also have a higher risk of malignancies, mainly hepatocellular carcinoma (31). Patients with hepatocellular carcinoma associated with cirrhosis frequently demand hospitalization due to several complications, such as sepsis, decompensated cirrhosis, tumor bleeding, and for the neoplasm treatment. Commonly employed in-hospital procedures in hepatocellular carcinoma patients comprise angiography with embolization, percutaneous ablative therapy, liver resection, and liver transplantation (32, 33). Hepatocellular carcinoma may promote portal vein compression or invasion, and systemic hypercoagulability (31). Consequently, several factors make hospitalized cirrhotic patients more susceptible to portal thrombosis. This explains why the included studies in this systematic review showed a higher risk for portal thrombosis compared with control groups.

Independently on which side of the clot-to-bleed spectrum the cirrhotic patient is sitting, the extremities of the spectrum are composed of highly complex patients, imposing prolonged length of hospital stay and high risk of mortality. Studies which could not identify differences in mortality risk, such as Aldawood et al. (25), Walsh et al. (24), and Bogari et al. (21), had low power of analysis, and a type-II error is likely.

This liability generates an enormous difficulty in proposing guidelines for therapeutic and prophylactic anticoagulation in hospitalized cirrhotic patients. In this context, before initiating any prophylactic or therapeutic anticoagulation plan, hospitalized cirrhotic patients should ideally be rigorously investigated for varices and portal hypertension. High-risk esophageal varices should be treated, and extra care should be taken in patients with portal hypertension and previous bleeding episodes (34). Once a clot has been established, anticoagulation therapy should ideally be initially managed in an intensive care unit for patients with cirrhosis. Extra care should be taken for monitoring bleeding and serum coagulation factors, as well as for the progression of thrombosis. Anticoagulation medication should be ceased as soon as any sign of bleeding appears. These patients should be managed in institutions with immediately available endoscopy services and interventional radiology, in the case of progression to massive pulmonary embolism or massive bleeding (35).

This current review has limitations. The high inter-study clinical heterogeneity precludes a quantitative synthesis (meta-analysis). The included studies comprised a high number of liver cirrhosis etiologies and different grades of disease severity. Besides, most of the included studies were retrospective and some of them were population-based. Population-based data may lack some details regarding patients’ management, including bleeding definition and bleeding severity and regarding anticoagulant medications usage (36, 37).

Future original studies are needed, and analysis should be grouped according to the stratification of liver disease severity, such as Child-Pugh and MELD scores. Anticoagulation prophylactic and therapeutic methods should be studied according to the position that hospitalized cirrhotic patient is positioned along the clot-to-bleed spectrum (38).

## Conclusion

Hospitalized cirrhotic patients are a heterogeneous group of patients who may present a higher risk for thrombosis and bleeding than non-cirrhotic patients. Besides, thromboembolism during hospitalization significantly deteriorates cirrhotic patients’ prognosis, making patient management much more challenging.

## Data availability statement

The original contributions presented in this study are included in the article/Supplementary material, further inquiries can be directed to the corresponding author.

## Author contributions

LC, FT, and VR-S: searching. DW, RP, and RM: extracting. LN, LD, and RM: statistical analysis; AT, LC-D’A, and WA: reviewing and editing. All authors contributed to the article and approved the submitted version.

## Footnotes

1
https://gradepro.org/
