# Supplementary material for: Venous thromboembolism in in-hospital cirrhotic patients: A systematic review
Source: Front Med (Lausanne). 2022 Nov 7;9:1027882. doi: 10.3389/fmed.2022.1027882 (PMC9676642; doi:10.3389/fmed.2022.1027882)
Supplement: Supplementary file 1 [file Table_1.pdf]

Author	Year	Design	N cirrhotic patients	Cause of liver disease	Male (%)	Age	Child-Pugh	MELD	Ascitis (%)	Variceal bleeding (%)	HCC (%)	Malignancy (%)	Previous VTE (%)	Acute infection (%)	Albumine (g/dL)	Platelets ($10^3/mm^3$)	INR
**Studies comparing cirrhotic vs. non-cirrhotic patients**																	
**Cirrhotic patients**																	
Al-Dorzi et al.	2013	Cohort	75	NI	49	59	NI	NI	NI	NI	NI	9	0	73	NI	115	2.2
Barba et al.	2018	Cohort	135832	NI	69	65	NI	NI	14	5	NI	16	NI	NI	NI	NI	NI
Enger et al.	2014	Matched-Cohort	15158	Hep C	60	56	NI	NI	NI	NI	NI	NI	7	NI	NI	NI	NI
Gulley et al.	2008	Cohort	963	Hep C, alcohol, cryptogenic, Hep B	68	50	8.2	NI	NI	NI	NI	NI	NI	NI	2.7	144	1.7
Ng et al.	2015	Matched-Cohort	2223	NI	66	58	NI	NI	NI	NI	NI	22	NI	NI	NI	NI	NI
Wu and Nguyen	2010	Cohort	649879	NI	62	58	NI	NI	NI	NI	NI	NI	NI	NI	NI	NI	NI
Yang et al.	2015	Cohort	1296	NI	36	53	NI	NI	NI	NI	NI	16	NI	40	NI	NI	NI
**Non-cirrhotic patients**																	
Al-Dorzi et al.	2013	Cohort	723	NA	69	49	NA	NA	NI	NI	12	NI	12	37	NI	258	1.3
Barba et al.	2018	Cohort	5618687	NA	51	73	NA	NA	<1	<1	11	NI	NI	NI	NI	NI	NI
Enger et al.	2014	Matched-Cohort	45473	NA	60	56	NA	NA	NI	NI	NI	NI	3	NI	NI	NI	NI
Gulley et al.	2008	Cohort	12405	NA	64	50	NA	NA	NI	NI	NI	NI	NI	NI	3.7	254	1.1
Ng et al.	2015	Matched-Cohort	22230	NA	53	60	NA	NA	NI	NI	13	NI	NI	NI	NI	NI	NI
Wu and Nguyen	2010	Cohort	575057	NA	39	54	NA	NA	NI	NI	NI	NI	NI	NI	NI	NI	NI
Yang et al.	2015	Cohort	193532	NA	52	53	NA	NA	NI	NI	9	NI	NI	15	NI	NI	NI

Supplementary Table 1. Baseline characteristics of the included studies. Studies comparing cirrhotic vs. non-cirrhotic patients. NI: Not informed; NA: Not applicable; HCC: Hepatocellular carcinoma; VTE: Venous thromboembolism
