# Supplementary material for: Venous thromboembolism in in-hospital cirrhotic patients: A systematic review
Source: Front Med (Lausanne). 2022 Nov 7;9:1027882. doi: 10.3389/fmed.2022.1027882 (PMC9676642; doi:10.3389/fmed.2022.1027882)
Supplement: Supplementary file 2 [file Table_2.pdf]

Studies comparing cirrhotic with VTE vs. cirrhotic without VTE																	
**Author**	**Year**	**Design**	**N cirrhotic patients with VTE**	**Cause of liver disease**	**Male (%)**	**Age**	**Child-Pugh**	**MELD**	**Ascitis (%)**	**Variceal bleeding (%)**	**HCC (%)**	**Malignancy (%)**	**Previous VTE (%)**	**Acute infection (%)**	**Albumine (g/dL)**	**Platelets (10^3/mm^3)**	**INR**
**Cirrhotic patients with VTE**																	
Aldawood et al.	2011	Case-control	6	Hep C, Hep B, cryptogenic	33	64	10.3	NI	NI	33	17	NI	NI	33	2.6	201	1.55
Ali et al.	2011	Case-control	8248	NI	59	58	NI	NI	26	6	NI	NI	NI	NI	NI	NI	NI
Barba et al.	2018	Cohort		NI	31	65	NI	NI	NI	2	NI	12	NI	NI	NI	NI	NI
Bogari et al.	2014	Cohort	18	NI	67	54	NI	17.8	NI	NI	NI	22	0	12	2.6	184	1.4
Gîrleanu et al.	2012	Case-control	78	NI	63	60	7.7	13.2	58	NI	NI	NI	NI	22	2.5	101	1.6
Lesmana et al.	2010	Case-control	12	Hep C, Hep B	12	NI	NI	NI	50	NI	33	NI	NI	NI	2.8	131	NI
Lizarraga et al.	2010	Matched Case-control	108	Viral, alcohol, autoimmune, NASH, hemochromatosis, cryptogenic	64	56	NI	NI	NI	NI	NI	NI	NI	NI	2.3	143	NI
Søgaard et al.	2009	Matched Case-control	99464	NASH, alcohol	47	NI	NI	NI	NI	NI	NI	17	NI	NI	NI	NI	NI
Stine et al.	2018	Matched Case-control	145	NASH, cryptogenic, Hep C, alcohol, autoimmune, cholestatic, Hep B, hemochromatosis	62	59	NI	15.7	69	53	11	12	22	47	2.9	153	1.5
Walsh et al.	2013	Matched Case-control	27	NI	NI	NI	NI	17	NI	NI	NI	NI	NI	NI	2.1	103	1.5
Zhang et al.	2016	Case-control	9	Hep B, alcohol	89	57	NI	12.4	88	NI	NI	NI	NI	NI	2.9	75	1.7
**Cirrhotic patients without VTE**																	
Aldawood et al.	2011	Case-control	220	Hep C, Hep B, cryptogenic, autoimmune	63	63	8.25	NI	NI	24	NI	45	NI	54	3	136	1.3
Ali et al.	2011	Case-control	441551	NI	61	58	NI	NI	31	9	NI	NI	NI	NI	NI	NI	NI
Barba et al.	2018	Cohort	318453	NI	34	65	NI	NI	3	<1	13	NI	NI	NI	NI	NI	NI
Bogari et al.	2014	Cohort	145	NI	65	54	NI	16.8	NI	NI	19	NI	1	57	2.6	124	1.5
Gîrleanu et al.	2012	Case-control	160	NI	63	55	7.9	10.3	49	NI	NI	NI	NI	7	3.2	109	1.5
Lesmana et al.	2010	Case-control	244	Hep B, Hep C, Alcohol, NASH	64	NI	NI	NI	50	NI	NI	34	NI	NI	3	148	NI
Lizarraga et al.	2010	Matched Case-control	108	Cryptogenic, viral, alcohol, NASH, autoimmune, hemochromatosis	64	56	NI	NI	NI	NI	NI	NI	NI	NI	2.4	109	NI

Søgaard et al.	2009	Matched Case-control	496979	NASH, alcohol	47	NI	NI	NI	NI	NI	6	NI	NI	NI	NI	NI	NI
Stine et al.	2018	Matched Case-control	145	NASH, cryptogenic, Hep C, alcohol, cholestatic, hemochromatosis, autoimmune	62	58	NI	16.3	69	51	14	12	3.5	31	2.9	115	1.6
Walsh et al.	2013	Matched Case-control	81	NI	NI	NI	NI	17	NI	NI	NI	NI	NI	NI	2.4	83	1.4
Zhang et al.	2016	Case-control	1997	Alcohol, Hep B, Hep C, autoimmune, cholestatic, drug	66	56	NI	7.4	49	NI	NI	NI	NI	NI	3.2	101	1.3

Supplementary Table 2. Baseline characteristics of the included studies. Studies comparing cirrhotic with VTE vs. cirrhotic without VTE. NI: Not informed; NA: Not applicable; HCC: Hepatocellular carcinoma; VTE: Venous thromboembolism
