# Supplementary material for: Venous thromboembolism in in-hospital cirrhotic patients: A systematic review
Source: Front Med (Lausanne). 2022 Nov 7;9:1027882. doi: 10.3389/fmed.2022.1027882 (PMC9676642; doi:10.3389/fmed.2022.1027882)
Supplement: Supplementary file 3 [file Table_3.pdf]

Certainty assessment						
Studies	Risk of bias	Inconsistency	Indirectness	Imprecision	Publication bias	Overall certainty of evidence
**LOS**						
3 observational studies	very serious[a]	very serious[b]	not serious	not serious	none	⊕◯◯◯ Very low
**VTE**						
7 observational studies	very serious[a]	very serious[b]	not serious	not serious	none	⊕◯◯◯ Very low
**Pulmonary embolism**						
4 observational studies	very serious[a]	very serious[b]	not serious	not serious	none	⊕◯◯◯ Very low
**Portal thrombosis**						
2 observational studies	very serious[a]	very serious[b]	not serious	not serious	none	⊕◯◯◯ Very low
**Bleeding**						
4 observational studies	very serious[a]	very serious[b]	not serious	not serious	none	⊕◯◯◯ Very low

Supplementary Table 3. GRADE (Grading of Recommendations, Assessment, Development and Evaluations). a. Bias in the selection of participants into the study or due to missing data; b. High clinical heterogeneity; LOS: Length of hospital stay; VTE: venous thromboembolism.
