# Supplementary material for: Venous thromboembolism in in-hospital cirrhotic patients: A systematic review
Source: Front Med (Lausanne). 2022 Nov 7;9:1027882. doi: 10.3389/fmed.2022.1027882 (PMC9676642; doi:10.3389/fmed.2022.1027882)
Supplement: Supplementary file 4 [file Table_4.pdf]

The risk of bias in non-randomized studies of interventions (ROBINS-I) assesmente toolfo cohort-type studies								
	1. Bias due to confounding	2. Bias in selection of participants into the study	3. Bias in classification of interventions	4. Bias due to deviations from intended interventions	5. Bias due to missing data	6. Bias in measurement of outcomes	7. Bias in selection of the reported results	8. Overall Bias
Al-Dorzi et al.	Low	Critical	Low	Low	Critical	Low	Low	Serious
Barba et al.	Low	Critical	Low	Low	Critical	Low	Low	Serious
Enger et al.	Low	Low	Low	Low	Critical	Low	Low	Moderate
Gulley et al.	Low	Critical	Low	Low	Moderate	Low	Low	Moderate
Ng et al.	Low	Low	Low	Low	Critical	Low	Low	Moderate
Wu and Nguyen	Low	Critical	Low	Low	Critical	Low	Low	Serious
Yang et al.	Low	Critical	Low	Low	Critical	Low	Low	Serious
Aldawood et al.	Low	Critical	Low	Low	Moderate	Low	Low	Moderate
Ali et al.	Low	Critical	Low	Low	Critical	Low	Low	Serious
Bogari et al.	Low	Critical	Low	Low	Moderate	Low	Low	Moderate
Gîrleanu et al.	Low	Critical	Low	Low	Moderate	Low	Low	Moderate
Lesmana et al.	Low	Critical	Low	Low	Critical	Low	Low	Serious
Lizarraga et al.	Low	Low	Low	Low	Critical	Low	Low	Moderate
Søgaard et al.	Low	Low	Low	Low	Critical	Low	Low	Moderate
Stine et al.	Low	Low	Low	Low	Moderate	Low	Low	Low
Walsh et al.	Low	Low	Low	Low	Moderate	Low	Low	Low
Zhang et al.	Low	Critical	Low	Low	Moderate	Low	Low	Moderate

Supplementary Table 4. Risk of bias assessment. The risk of bias in non-randomized studies of interventions (ROBINS-I) assesmente toolfo cohort-type studies
